# Hunter–Gatherer children's close-proximity networks: Similarities and differences with cooperative and communal breeding systems

**DOI:** 10.1017/ehs.2024.1

**Published:** 2024-01-31

**Authors:** Nikhil Chaudhary, Abigail E. Page, Gul Deniz Salali, Mark Dyble, Daniel Major-Smith, Andrea B. Migliano, Lucio Vinicius, James Thompson, Sylvain Viguier

**Affiliations:** 1 Leverhulme Centre for Human Evolutionary Studies, Department of Archaeology, University of Cambridge, Cambridge CB2 1QH, UK; 2 Department of Anthropology, University College London, London WC1H 0BW, UK; 3 Department of Population Health, London School of Hygiene and Tropical and Medicine, London WC1E 7HT, UK; 4 Population Health Sciences, Bristol Medical School, University of Bristol, Bristol BS8 2BN, UK; 5 Department of Anthropology, University of Zurich, 8057 Zürich, Switzerland

**Keywords:** Hunter–gatherers, cooperative breeding, allomothering, childcare, cooperation

## Abstract

Among vertebrates, allomothering (non-maternal care) is classified as cooperative breeding (help from sexually mature non-breeders, usually close relatives) or communal breeding (shared care between multiple breeders who are not necessarily related). Humans have been described with both labels, most frequently as cooperative breeders. However, few studies have quantified the relative contributions of allomothers according to whether they are (a) sexually mature and reproductively active and (b) related or unrelated. We constructed close-proximity networks of Agta and BaYaka hunter–gatherers. We used portable remote-sensing devices to quantify the proportion of time children under the age of 4 spent in close proximity to different categories of potential allomother. Both related and unrelated, and reproductively active and inactive, campmates had substantial involvement in children's close-proximity networks. Unrelated campmates, siblings and subadults were the most involved in both populations, whereas the involvement of fathers and grandmothers was the most variable between the two populations. Finally, the involvement of sexually mature, reproductively inactive adults was low. Where possible, we compared our findings with studies of other hunter–gatherer societies, and observed numerous consistent trends. Based on our results we discuss why hunter–gatherer allomothering cannot be fully characterised as cooperative or communal breeding.

## Graphical abstract

**Figure:**
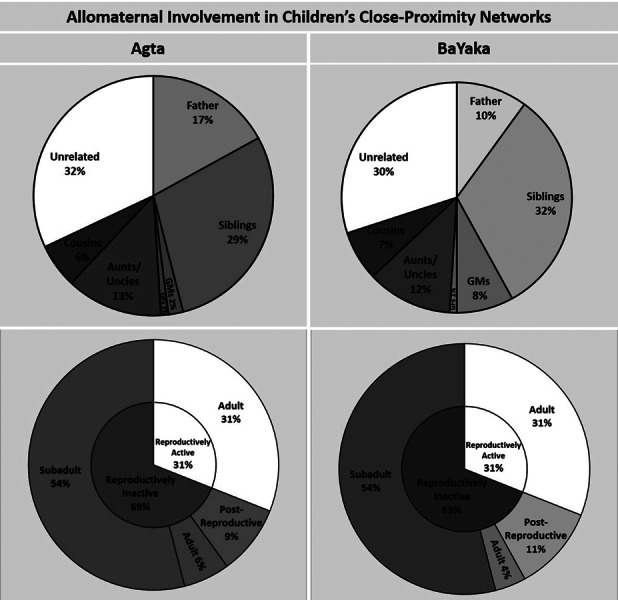

**Social media summary:** Are we really cooperative breeders? Hunter–gatherer cooperation in childcare is unique from a cross-species perspective.

## Introduction

Human reproduction is remarkably efficient, with women in natural fertility populations producing highly dependent large-brained offspring at higher fertility and survival rates than other apes (Kramer, 2010). It is well established that rearing multiple expensive offspring simultaneously is facilitated by extensive assistance from non-maternal helpers or ‘allomothers’ (Hrdy, 2009; Sear & Mace, 2008). Allomothering occurs in a diverse range of taxa and takes many forms such as food provisioning, babysitting, transporting and incubation (Clutton-Brock, 2002; Griesser et al., 2017; Riehl, 2013). Among vertebrates, it is classified into systems of cooperative or communal breeding according to whether helpers are reproductively active, and the underlying evolutionary mechanisms (see next section) (Burkart et al., 2017; Lukas and Clutton-Brock, 2012a). Humans have been described with both labels, and very frequently as cooperative breeders (Hrdy, 2007; Kramer, 2010; Wu et al., 2013). However, numerous scholars have challenged the applicability of these terms to our species (Bogin et al., 2014; Burkart et al., 2017; Strassmann and Kurapati, 2010). Burkart *et al.* (2017: 1) emphasise that to understand evolutionary pathways to cooperation we must avoid “looking for unity in diversity”. Therefore, it is important to establish the similarities and differences between allomothering across species, including humans. Below we outline how non-human allomothering systems are classified, we then briefly review existing research on hunter–gatherer allomothering, highlight obstacles to its classification, and explain how we aim to address these in the present study of Agta and BaYaka hunter–gatherers.

### Classification of allomothering systems

#### Cooperative breeding

The specificity of criteria for the classification of cooperative breeding varies across the literature. A liberal definition is that it refers to social systems in which non-breeding allomothers help a breeding female or a male–female breeding pair in raising offspring (Burkart et al., 2017). However, most researchers restrict the use of the term to cases in which non-breeding allomothers are sexually mature, which begs the question of why they would remain non-reproductive and help another female raise her offspring (Emlen, 1982; Lukas & Clutton-Brock, 2012b); we refer to this as the traditional definition. This occurs in less than 5% of mammals (Burkart et al., 2017; Lukas & Clutton-Brock, 2016), and approximately 10% of bird species (Cockburn, 2006).

The dominant explanation for cooperative breeding is that owing to ecological constraints on personal reproduction, e.g. low availability of suitable nesting sites, or benefits of philopatry, e.g. reduced predation, offspring delay dispersal from their natal group and become ‘helpers at the nest’ (Arnold & Owens, 1998; Emlen, 1982; Koenig et al., 1992). There, they gain indirect fitness benefits by helping their parents raise the next brood/litter (Cornwallis et al., 2009; Griesser et al., 2017; Hatchwell, 2009; Lukas & Clutton-Brock, 2012a). Allomothering evolves when the benefits that can be conferred to breeders are high, for example in unpredictable/harsh environments as seen in meerkats and babbling starlings, or in species with large litter sizes such as naked mole rats (Griesser et al., 2017; Lukas & Clutton-Brock, 2012b, 2016; Rubenstein & Lovette, 2007).

Mechanisms beyond kin selection have been shown to operate in some, but substantially fewer, cooperative breeders (Kokko et al., 2002; Piper et al., 1995; Sherley, 1990). However, numerous phylogenetic analyses have demonstrated that the evolution of cooperative breeding requires high relatedness between potential providers and recipients of allomothering, and as such it is restricted to socially monogamous species with low promiscuity (Cornwallis et al., 2009; Lukas & Clutton-Brock, 2012a). Other drivers of helping may be supplementary to kin selection or contribute to the maintenance of the system once it has evolved.

#### Communal breeding

Communal breeding occurs when multiple breeding females, or in some cases multiple breeding male–female pairs, share care and/or provisioning responsibilities (Lukas & Clutton-Brock, 2012a). This system is observed in a small minority of mammals, such as the banded mongoose, and birds such as the greater anis (Gilchrist et al., 2004; Pusey & Packer, 1994; Riehl, 2013).

The evolution of these systems does not rely on kin selection; co-breeding females may be unrelated and allomothering is often driven by reciprocity or mutualism (Baden et al., 2013; Bebbington et al., 2018). Mothers often crèche their young together. This can provide greater protection from predators or infanticide, as seen in African lions, or allow mothers to increase foraging time if they take turns in caring for or feeding the crèche, as observed in free-living house mice (Auclair et al., 2014; Packer et al., 2001). Communal breeding can also increase foraging efficiency, for instance female sperm whales share information and engage in cooperative hunting (Rendell et al., 2019).

In summary, the most fundamental difference between cooperative and communal breeders is that allomothers are non-breeders in the former and breeders in the latter. This distinction is very important as it is only in cooperative breeding that it is necessary to explain why sexually mature allomothers do not reproduce even though they are physiologically capable of doing so. Accordingly, the evolution of cooperative breeding relies on kin selection. This is not the case in communal breeders as cooperation between unrelated individuals is frequent and driven by reciprocity and mutualism. Thus, identifying allomothers’ reproductive status and relatedness to the recipients of care is key for characterising an allomothering system.

Humans are frequently labelled as cooperative breeders since allomothering is normative in natural fertility populations (Burkart et al., 2009; Hrdy, 2007). However, many researchers would agree that allomothering in the vast majority of human societies does not neatly fit the traditional definition of cooperative breeding. The purpose of inter-specific classification schemes is to identify similarities across taxa which can then aid in uncovering fundamental evolutionary processes. Here, we aim to describe hunter–gatherer allomothering systems in a way that highlights the extent to which they resemble and differ from cooperative and communal breeding systems.

### Hunter–gatherer allomothering

Given that our species lived as hunter–gatherers for the majority of our evolutionary history, studying contemporary hunter–gatherers can be informative for our understanding of the evolution of human allomothering systems. We emphasise that contemporary hunter–gatherers are modern populations who, just like any other extant human population, have undergone millennia of their own biological and cultural evolution; they are not ‘living fossils’ (Kelly, 2013). It is only because their mode of subsistence overlaps with that of ancestral populations that they can offer insight into human prehistory.

Much of the hunter–gatherer research has focused on the behaviour of fathers or post-reproductive grandmothers, emphasising their role as the primary allomother (Hawkes et al., 1998, 2014; Marlowe, 2003; Wood & Marlowe, 2014). The traditional pair-bonding model argues that biparental care was fundamental for child survival over our species’ evolutionary history (Lovejoy, 1981), and highlights that in many foraging societies males produce the majority of calories (Hill & Hurtado, 1996; Kaplan et al., 2000; Marlowe, 2003; Wood & Marlowe, 2014). Fathers can also be important providers of hands-on childcare, for instance paternal care among Aka foragers is the highest recorded in any human society (Hewlett, 1991). However, the role of fathers is contested and male hunting has been argued to be a form of ‘showing off’ phenotypic quality rather than paternal investment (Hawkes et al., 2001, 2014).

Many anthropologists have instead emphasised the primary importance of grandmothers as allomothers. For instance, Hadza grandmothers spend an extensive amount of time foraging, and their foraging effort is positively associated with their grandchildren's health (Hawkes et al., 1997). Similarly, Martu aborigines are described as occupying a ‘grandmaternal niche’, and grandmothers provide considerably more childcare than any other allomother (Scelza, 2009). Grandmothers may also confer benefits via their ecological knowledge and expertise. For example, we found that among the BaYaka, medicinal plant knowledge increases with age and tends to be shared within families (Salali et al., 2016, 2020). Some scholars also endorse the grandmother hypothesis, which argues that the inclusive fitness benefits of grandmothering are sufficiently large to have driven the evolution of the post-reproductive lifespan (Hawkes & Coxworth, 2013).

The contribution of juveniles has also been studied, and some similar trends have been reported in several societies (Helfrecht et al., 2020; Ivey, 2000; Kramer, 2005; Page et al., 2021). Older children may supervise young in the context of mixed-age ‘playgroups’ (Lew-Levy et al., 2017; Page et al., 2021; Salali et al., 2019). Juveniles also forage for foods such as fruits and plants which they have sufficient strength and skill to acquire (Crittenden et al., 2013; Salali et al., 2019); this can help with provisioning when they target foods that adults do not (Kramer, 2014). Finally, they often play an important role in food processing, domestic tasks and collecting firewood and water; and in turn, mothers are able to engage in other activities (Hawkes et al., 1997; Lew-Levy et al., 2022).

### Requirements for classification

Studying the behaviour of a specific single category of allomother – such as fathers, grandmothers, or juveniles – when they are co-residing with a child overlooks the frequency with which they are actually living with an average child and also other potential sources of care. It is necessary to account for the presence and number of individuals in each category of potential allomother, i.e. how many individuals there are in each category living in the same camp as a child; herein, we refer to this as *availability*. For instance, if an average co-resident sibling provides much more care than an average unrelated campmate, we might infer that the allomothering system is largely driven by kin selection and resembles a ‘helpers at the nest’ system. However, if the average child has many more unrelated campmates than siblings, it may be that the aggregate contribution of all unrelated campmates combined is greater than that of all siblings combined. In this case the aforementioned inference would be misleading. Therefore, the taking of a child's-eye perspective, rather than that of any specific category of allomother, is required for charactering the allomothering system as a whole, and in turn for inter-specific comparison and classification.

Some child's-eye studies of allomaternal networks have been conducted (Helfrecht et al., 2020; Marlowe, 2005b; Scelza, 2009). However, comparing hunter–gatherer allomothering with cooperative and communally breeding species requires identifying the relative contributions of all allomothers according to their (a) reproductive status and (b) relatedness to the recipients of care.

1. Few studies quantify the relative childcare contributions of subadult (not just siblings), adult, and post-reproductive (not just grandmothers) helpers; see Helfrecht et al. (2020) for an exception. We know of no study which presents all of these data and distinguishes between reproductively active and reproductively inactive adults, here we are using the term adult to refer to individuals who are of reproductive age. Reproductively inactive adults may constitute a non-trivial constituent of a child's allomaternal network (Ivey, 2000); and importantly, it is only their help that adheres to the traditional definition of cooperative breeding. Equally, communal breeding is defined by sharing of care among reproductively active adults, not adults in general.
2. There are numerous studies highlighting that food transfers between unrelated campmates are important for reducing unpredictability in resource access, and for provisioning reproductively active women. This has been demonstrated in African, Asian and South American hunter–gatherers (Dyble et al., 2016; Gurven et al., 2000; Hill & Hurtado, 2009). Surprisingly, very few studies have focused on the childcare contribution of unrelated helpers. Helpers who are not fathers, grandmothers or siblings are often lumped together as ‘other helpers’, therefore it is not possible to isolate the contribution of unrelated campmates. Examining the relative contribution of related and unrelated helpers is important for inter-specific comparison since the evolution of cooperative, but not communal, breeding is associated with high relatedness between potential allomothers and young. Existing childcare studies in which data on the contribution of unrelated campmates are presented, or can be calculated, are included in a summary table in our Results section (Table 6).

### The current study

Here we examine the allomothering systems of two hunter–gatherer societies – the Agta from the Philippines and the BaYaka from Congo. We used remote-sensing devices (motes) to measure the amount of time potential allomothers spend in close proximity to infants and toddlers. Our fundamental aim is to characterise children's close-proximity networks in a manner that adds to our understanding of how hunter–gatherer allomothering resembles/differs from cooperative and communal breeding systems. As such, we take a child's-eye perspective and quantify the time spent in close proximity to all categories of potential allomother. Categories reflect potential allomothers’ reproductive status, and their relationship with a child including whether they are related or unrelated. Importantly, we quantify their involvement in the close-proximity networks at the individual and aggregate (time spent by *all* members of the category) levels, to account for helper availability.

#### The role of close proximity in allocare systems

Allomothering is typically divided into direct and indirect *investment*. The former is also referred to as allocare and includes all care that requires close proximity to an infant, and the latter to other forms of help such as territory defence or resource provisioning (Kleiman & Malcolm, 1981). Studies of direct investment, herein *childcare*, often distinguish between care that is high cost/demand/investment vs. low cost/demand/investment. The specific term used varies by study; however, the distinctions being made are similar. High investment care involves focussed attention and interaction such as feeding or grooming, whereas low investment care does not necessarily require interaction or much energy expenditure e.g. supervision (Meehan, 2005; Scelza, 2009). Proximity is frequently measured in anthropological studies of childcare since it is a requirement for both high and low investment childcare (Hewlett & Lamb, 2009; Konner, 2018; Marlowe, 2005b; Page et al., 2021). It is sometimes highlighted as a form of low investment care in and of itself since it facilitates supervision of a child and the potential to intervene if required (Meehan et al., 2013, 2018; Page et al., 2019). In turn, it provides greater opportunity for mothers to rest or engage in labour activities, such as foraging or food processing, without putting their child at risk (Page et al. 2019). Indeed, this is one of the primary functions of what we call ‘babysitting’ in industrialised societies. Ethologists have noted that even when babysitting involves little active or costly care, it can confer major benefits in communally breeding species (Burkart et al., 2017; Lewis & Pusey, 1997).

The motes provide valuable high-resolution data on the time each potential allomother spends in close proximity to a child. However, it is important to acknowledge that we cannot discern the amount of high investment care a potential allomother may be providing, nor how much of the time they actually spend intervening as opposed to simply being in a position to intervene. In the Methods section we discuss the benefits and limitations of the motes in more detail.

We predict that Agta and BaYaka children rely on many allomothers, differing in reproductive status and relatedness, rather than any single category of helper; and as such, hunter–gatherers cannot be classified as communal *or* cooperative breeders. We expect that those categories of potential allomother who are the most available spend the most time in close proximity to children at the aggregate level. Different categories of helpers are available as a result of different forces – such as life-history schedules and residence patterns – some of which have parallels with the forces producing helper availability in cooperative and communally breeding non-human animals; we consider these in our discussion. Accordingly, by identifying the involvement of different helpers, it is possible to comment on the extent to which hunter–gatherer allomothering resembles and differs from cooperative and communal breeding systems.

## Methods

This study has approval from the Ethics Committee of University College London (UCL Ethics code 3086/003), and the methods were carried out in accordance with the approved guidelines. Research permission was granted by the Republic of Congo's Ministry of Scientific Research and local government in the Philippines. Data were collected between February and October 2014. Informed consent was obtained for all participants. The purpose of the study and what it would entail was explained to all camp members in the local language by translators. They were then asked if they were interested in participating. Translators then read a prepared consent form to all potential participants in the local language, which willing participants signed via a pen mark or thumbprint according to their preference. If a child was under the age of 18, consent could be provided by either one of their parents. There was one child who lived in a household with her grandparents because both of her parents had moved to the city for labour opportunities; in her case, consent was provided by her grandmother who was a socially recognised guardian. Participating households were compensated with a gift of their choice out of thermal bottles, cooking pans and utensils or tools.

### Study populations

The Agta and BaYaka are best described as *immediate-return* hunter–gatherers. This is an anthropological term referring to hunter–gatherer societies that do not accumulate material resources or store food and have mobile residence patterns and an egalitarian political organisation (Woodburn, 1982). Allomothering is frequent in both populations (Page et al., 2017, 2021).

#### The Agta

The Agta communities that we worked with live in the coastal Palanan region of northeast Luzon, the Philippines. The average camp size is seven households and just under 50 individuals, average relatedness within camps is low and mobility between camps is high (Dyble et al., 2015; Smith et al., 2019). Relationships are serially monogamous and polygyny does not occur. The Agta are bilocal, meaning that a couple is equally as likely to live with the man's relatives as the woman's relatives. However, it appears that Agta families that include young children may be more likely to stay with the mother's relatives. We found that nine of the Agta children in this study were living in the same camp as their maternal grandmother but only three were living with their paternal grandmother.

The communities we worked with rely heavily on spearfishing, inter-tidal foraging and the gathering of wild foods, and only occasionally hunt terrestrial game. Their foraged foods are supplemented by trading with local agricultural groups and in some cases wage labour (Smith et al., 2017; Dyble et al., 2019). Hunting is a male activity despite famous reports of women hunting in neighbouring Agta populations (Goodman et al., 1985). Boys spend more time hunting as they get older, usually in groups of similar-aged adolescents. Spearfishing is also predominantly, although not exclusively, done by men, especially in open-water or in rivers that are distant from camp. Subadults of both sexes may spearfish, but there is then a decline among females after they have dependent offspring. Agta of all ages and both sexes engage in inter-tidal foraging for a variety of resources including octopus and shellfish; these trips often occur in family groups led by the mother. While gathering wild resources is predominantly a female activity, it also often occurs in family groups which sometimes include adult men. In summary, there are several economic activities that are at least sometimes carried out by both sexes and across age-groups. Nevertheless, the division of labour is still structured in a manner that affects opportunities for childcare. Infants and toddlers are often present when women or subadults are gathering or fishing. Although men are sometimes present on these expeditions, they also frequently go spearfishing, and occasionally hunt, without women or children.

As infants begin to rely less on their mothers’ milk they spend an increasing amount of time in mixed-age and mixed-sex playgroups, and by 2 years of age this constitutes a significant proportion of their day. These playgroups are child-only and represent a key context in which subadults are responsible for supervision and providing other forms of care. Children of both sexes engage in childcare, but girls are usually more involved. In an experimental game assessing Agta children's cooperative behaviour, we found that older children willingly shared food resources with younger children (Major-Smith et al., 2023).

#### The BaYaka

We worked with BaYaka hunter–gatherers who live in the Ndoki rainforest in the Republic of Congo. Camp size varies from 20 to 80 individuals, average genetic relatedness within camps is low, and there is high between-camp mobility (Dyble et al., 2015). As with the Agta, BaYaka residence is generally bilocal but families with young children appear to be more likely to live with the mother's relatives. Eleven of the children were living in the same camp as their maternal grandmother, but only three were living with their paternal grandmother. Relationships are predominantly serially monogamous; there are cases of polygyny but the point-prevalence is below 10% (Chaudhary et al., 2015). In the cases where a man has more than one wife, the wives reside in different camps and the man splits his time between camps.

Unlike some Central African foragers, only men engage in hunting and trapping in the communities we worked with. After reaching adolescence, boys may begin to accompany their fathers or other older men on these expeditions. It is also only men who climb trees to collect honey, although the whole family may go on these trips. Females contribute to subsistence via fishing as well as gathering yams and wild plants, and their foraging expeditions frequently occur in groups including both adult women and girls. Finally, children of both sexes often collect fruits and mushrooms when in playgroups. Infants and toddlers may be taken along for all foraging activities except for hunting and trapping; therefore foraging and childcare are often mutually exclusive activities for adult men but not for women or subadults. The BaYaka also engage in some trade with members of neighbouring Bantu communities, exchanging wild foods for alcohol, cigarettes or manioc (Chaudhary et al., 2016; Knight et al., 2021).

Both girls and boys become involved in childcare from around the age of 4, although the involvement of young girls rises rapidly relative to boys and their contribution is larger throughout the rest of the subadult period (Salali et al., 2019). It is not uncommon to see very young children of both sexes feeding, soothing and carrying infants, and a video analysis of a 4-year-old boy suggested that children are capable of sensitive caregiving from a very young age (Mesman et al., 2018). Another study found that girls between 4 and 7 years of age increase the foraging efficiency of nursing women during group expeditions by looking after their infants (Jang et al., 2022). As with the Agta, older infants and toddlers spend an increasing proportion of time in mixed-age child-only playgroups from around the age of 2, in which older children are responsible for their supervision and care.

It is noteworthy that this research was conducted with an ethnolinguistic subgroup of the BaYaka who speak Mbendjele. They are distinct from the Aka, who are a different BaYaka subgroup living in the Central African Republic. To avoid confusion, we explicitly refer to the latter as the Aka when making references to research that has been conducted with them.

### Data collection

There were no exclusion criteria for this study since we wanted to provide a characterisation of children's close-proximity networks that was as complete and representative as possible. All camp residents were free to participate, and they were all willing to do so. In both populations close-proximity networks were constructed for all children under four years of age. The study sample was comprised of six Agta and three BaYaka camps, and we constructed close-proximity networks for 49 children between 0.08 and 3.99 years of age. It included a total of 30 Agta children, including one set of twins, and 890 allomother–child dyads; and 19 BaYaka children, none of whom were twins, and 790 allomother–child dyads. In the case of two BaYaka children and one Agta child, the exact relationship with all campmates could not be discerned owing to missing information, and as such some analyses have slightly smaller sample sizes. Therefore, we specify samples sizes throughout the Results section.

### Measuring close proximity

To quantify the relative amount of time each potential allomother spent in close proximity to each infant/toddler under 4 years of age we used motes, which are portable remote sensing devices; more specifically, we used the UCMote Mini with a TinyOS operating system. They are programmed to emit an imperceptible radio signal known as a ‘beacon’ at a specified time interval, which is then received and stored in the internal memory of any other mote within a specified distance of the sender mote. Upon receipt of a beacon, the recipient mote stores an identification number corresponding to the sender mote.

All camp members wore a mote with a unique identification number in adjustable belt pouches/wristbands for between 5 and 9 days depending on the camp. We programmed all motes to emit beacons every 2 minutes during this period and to record any beacons registered within a 3 meter radius. We chose 3 metres since an allomother can pay clear attention to the child within this distance and quickly intervene if required; this threshold has been used as a measure of proximity in observational studies of hunter–gatherer childcare (e.g. Marlowe, 2005b; Page et al., 2021).

By downloading the data from the internal memory of all the motes it was possible to construct children's *close-proximity networks*. These quantify the relative proportion of time each child spends in close proximity to each potential allomother. The networks are complete since all individuals a child could interact with were wearing motes. Only beacons recorded between 05:00 and 20:00 were included to prevent data reflecting which individuals slept in the same shelter. For the Agta, we also have data derived from setting the motes threshold to 1.5 metres in five of the six camps. We characterised children's close-proximity networks at this threshold as a sensitivity analysis and the key trends remained the same; these results are available in the supplementary material (Figures S1 and S2).

The transition from being cared for to becoming a source of care is extremely fast and seamless; therefore, we considered all individuals older than 4 years of age as potential allomothers. Some children were observed to have transitioned from receiving care to providing it on research trips that were less than a year apart; this is usually catalysed by the birth of a younger sibling whom they begin to care for. The average inter-birth interval among hunter–gatherers is ~3.5 years (Marlowe, 2005a; Pennington, 2001). In our entire sample of potential allomothers (*n* = 283), there were only three individuals under the age of 7.5 – an age at which children unequivocally contribute to childcare – who did not have a younger sibling in camp. Had we chosen a higher age threshold for classifying an individual as a potential allomother, we would have missed the very important contribution of young children in caring for their even younger siblings. Furthermore, as described above, a detailed video analysis of caregiving by a 4-year old BaYaka boy suggested that children can make valuable contributions to childcare from this age (Mesman et al., 2018).

#### Advantages and limitations of the motes

In the supplementary material we present a verification of the motes data using data from a smaller sample of observational focal follows. The verification indicates that the close proximity being measured by the motes does indeed reflect the amount of time potential allomothers are in position to watch over a child and intervene if required. Some of this time may involve actual intervention and high investment care, but it is not possible to determine how often, if at all, this occurs. Thus, we can only conclude that at a *minimum* a potential allomother is in a position where they can watch over a child and intervene if required, but more costly care may also be provided. Given these limitations, our results do not offer a precise valuation of the allomaternal involvement of different helpers, and cannot be considered to be a complete description of children's allomothering networks.

Furthermore, food provisioning is a fundamental component of allomothering among hunter–gatherers and many other species. The motes do not offer insight into how much different allomothers contribute to provisioning. This limits the possibility of using the them in isolation to draw any firm conclusions regarding the extent to which hunter–gatherer allomothering resembles cooperative or communal breeding systems.

Despite these limitations on what can be inferred from the motes relative to traditional observational methods, they also offer some advantages. As mentioned, many observational studies of childcare also measure proximity, and sometimes categorise it as a form of low-cost care in and of itself. Observational studies often include ~15 focal children who are directly observed over the course of 12 daylight hours each (Meehan, 2005). These studies do produce substantial datasets; however, using the motes, it is possible to gather data from larger samples spanning longer periods of time since the researcher does not constantly need to be present after set-up. Here we present motes data for almost 50 children spanning between 5 and 9 days each. Additionally, in observational studies, data are only collected when there is daylight since the researcher needs to be able to see clearly (Ivey, 2000; Marlowe, 2005b; Meehan, 2005; Scelza, 2009). However, in our experience, hunter–gatherer communities often wake up before the sun has fully risen and do not go to sleep as soon as it gets dark. The motes do not require light and therefore can record data during these times. Thus, mote data are less likely to be subject to biases resulting from small sample sizes, short observation periods and a lack of data on care that occurs at certain times of day. In this sense, the motes are complementary to in-depth observational studies which offer high-resolution data on the specific caring behaviours provided by allomothers.

### Determining relationship type and relatedness between children and potential allomothers

We conducted genealogical and reproductive history interviews with all adults in each camp. Participants were asked to provide the name and sex of their grandparents, parents, parents’ siblings, siblings, offspring and spouse(s), including those that were deceased. Once this data was collected it was possible to determine the relationship type, e.g. grandparent–grandchild/cousin–cousin/unrelated etc., and the coefficient of relatedness between each child and potential allomother. Given that we are interested in examining the effects of relatedness, individuals are only categorised as aunts or uncles if they are biologically related to the child. ‘Unrelated’ refers to dyads with a coefficient of relatedness of *r* < 0.125.

### Assigning age

Age estimates were calculated for all members of the study population by creating relative age lists with the communities, as well as potential age ranges for all participants. The age ranges were based on dental development, whether their birth happened before or after a memorable event with a known date and sibling birth orders. This information was then integrated using a Gibbs sampling framework producing probability distributions for the age of each participant; these were then collapsed into point estimates based on the mean. For full information and a validation of this method see Diekmann et al. (2017).

### Characterising children's close-proximity networks

For each child we constructed a close-proximity network which is constituted of the time spent in close proximity with each potential allomother. We quantify the *involvement* of each potential allomother in each child's close-proximity network. We do this by calculating the amount of time that a given child spends in close proximity to a given potential allomother as a proportion of the time that child spends in close proximity to all potential allomothers. This is simply equivalent to the number of beacons received by a child's mote from a potential allomother's mote as a proportion of the total number of beacons received by that child's mote from the motes of all potential allomothers.

To compare the involvement of different categories of potential allomothers, e.g. fathers, unrelated individuals etc., we calculated the *aggregate* and *adjusted involvement* of each category of allomother. For each child, the aggregate involvement of a given category of potential allomother is calculated by summing the involvement of all members of that category in the child's network. We sum them because a complete characterisation of an allomothering system must incorporate the fact that some categories of helper are more available, i.e. have more members in a camp than others.

We reiterate the importance of this measure using an example given in the introduction. If an average co-resident sibling helps much more than an average unrelated campmate, we may infer that the allomothering system resembles a sibling helpers at the nest system and is underpinned by kin selection. However, it may be the case that children often do not have any siblings but usually have many unrelated campmates, and consequently the *aggregate* involvement of siblings is much lower than that of non-kin. In this case the inference about kin selection and sibling helpers at the nest would have been misleading.

The purpose of the *adjusted* involvement measure is to facilitate comparison of the involvement of an average individual of each category *when they are available*, i.e. alive and co-residing with a child. Therefore, only cases in which at least one individual of a given category is available to a child are incorporated into the calculation. For instance, if a child's father is deceased or lives in another camp, the lack of involvement of that father is irrelevant to understanding what fathers do when they are available; therefore, it is ignored in the calculation. For a given child, the adjusted involvement of a category is the mean involvement of members of that category.

In combination with aggregate involvement, adjusted involvement offers insight into the dynamics of the allomothering system at a lower-level and how it is related to availability. For example, fathers may be highly involved when they are available, but the majority of children may not have access to their fathers owing to high male mortality. In this case, the aggregate involvement of fathers would be low, but the adjusted involvement would be high since the latter only considers how involved fathers are in cases when they are present. Providing both the aggregate and adjusted involvement of different categories allows for a more complete description and nuanced understanding of children's close-proximity networks. Table 1 provides definitions of these measures for reference, and a worked example is available in Table S1.

**Table 1.** Glossary of key measures

Measure	Definition	Calculation
*Involvement* (of a potential allomother)	The amount of time that a child spends in close proximity to a given potential allomother as a proportion of the time that child spends in close proximity to all potential allomothers.	Beacons received by child's mote from the potential allomother's mote/total beacons received by child's mote from all potential allomothers’ motes.
*Aggregate involvement* (of a category of potential allomother)	The amount of time a child spends in close proximity to all members of a given category as a proportion of time that child spends in close proximity to all potential allomothers.	Sum of involvement of all members of a given category of potential allomother in a child's network.
*Adjusted involvement* (of a category of potential allomother)	The amount of time a child spends in close proximity to an average available member of a given category as a proportion of the time that child spends in close proximity to all potential allomothers. Available refers to cases when a potential allomother lives in the same camp as a child.	Mean involvement of a member of a given category of potential allomother in a child's network. Cases where there are zero available members in a given category are therefore excluded.

#### Categories of potential allomother

1. We calculated the aggregate and adjusted involvement of potential allomothers for the following relationship categories: fathers, siblings, grandmothers, grandfathers, aunts/uncles, cousins and unrelated campmates.
2. We also calculated the adjusted involvement of potential allomothers according to their level of relatedness to a child, which can take the values of: *r* = 0.5, *r* = 0.25, *r* = 0.125 and *r* =  0. Children often have more than 20 campmates with whom they are unrelated, which is far more than the number of potential allomothers they have at any other level of relatedness. Involvement may be highly skewed in such a large category. For instance, it may be the case that the majority of unrelated campmates are not particularly involved in a child's proximity network, but a few are heavily involved. Therefore, we separately calculated the involvement of each of the six most involved unrelated campmates to gain an insight into the level of skew. We also present data on the most involved potential allomother at each of the other levels of relatedness.
3. We calculated the aggregate and adjusted involvement of potential allomothers from each life-stage – subadults, adults and post-reproductive individuals. Again, we also calculate the involvement of the most involved potential allomother in each life-stage category.

We define subadults as individuals under 17 years old, which is the mean age of menarche among Agta females (Goodman et al., 1985); we apply this threshold in both populations because there is not an equivalent estimate for the BaYaka. There are no individuals in either sample under the age of 17 who have become parents. In both populations it is also rare for individuals to form a household with a spouse prior to reaching the age of 17. Post-reproductive stage individuals are females older than 50 years old, and males over 50 years old except those with a wife under 50. All other individuals are categorised as being in the adult, i.e. reproductive-age, life-stage. Categorising potential allomothers into these life-stages allows us to isolate the involvement of those individuals who are capable of personal reproduction; in other species, this is typically a prerequisite for allomothering to be classified as cooperative breeding.

Additionally, individuals are further classified as reproductively active or inactive. All subadults and post-reproductive individuals are categorised as reproductively inactive. Adults are considered reproductively active unless one of the following conditions is met, in which case they are considered reproductively inactive: (1) they have not had a live birth in the previous seven years of adulthood, which is approximately twice the average inter-birth interval among extant hunter–gatherers (Marlowe, 2005a; Pennington, 2001); and (2) they are living in their natal household and have had no live births, i.e. they have delayed ‘dispersal' and reproduction. In communal breeding systems allomothers are reproductively active, whereas in cooperative breeding systems they are reproductively inactive.

#### Children's age-groups

We provide a comparison of how our results vary for children under 2 years of age vs. those 2 years old and above. From approximately 2 years old, children spend a substantial amount of time in child-only playgroups during which they are in proximity to other children rather than adults, this represents a key transition in the composition of their proximity networks. This change in children's social life has been reported in many hunter–gatherer populations including the Agta and BaYaka (Chaudhary & Swanepoel, 2023; Lew-Levy et al., 2017; Page et al., 2021).

### Statistical analysis

The following analyses were conducted in *R version 4.2.1* and figures were created using the *ggplot2* package.

First, we wished to establish the shared features of the composition of children's proximity networks across the two populations as we were interested in identifying which elements of allomothering systems are the most consistent/variable across hunter–gatherer societies. Additionally, we wished to investigate how the composition of children's proximity networks may change with age. Many studies of direct care focus on early infancy. However, opportunities for allomothering probably increase as a child relies less on their mother's milk. Correspondingly, some studies highlight that around the age of 2 children begin spending more time in playgroups away from their mother (Lew-Levy et al., 2017; Page et al., 2021).

We used Dirichlet regressions to test for differences in the composition of children's proximity networks between the two populations and also between the two age-groups of children. Dirichlet regressions are appropriate when the dependent variable is compositional in nature, i.e. comprising multiple proportions that sum to 1.

We conducted one Dirichlet regression in which the dependent variable was the composition of a child's network in terms of the aggregate involvement of each relationship category of potential allomother, e.g. fathers, siblings, etc. We conducted another Dirichlet regression in which the dependent variable was the composition of a child's network in terms of the aggregate involvement of each life-stage category of potential allomother – subadults, adults and post-reproductive campmates. Since the dependent variable reflects the composition of a network, these analyses are conducted at the child-level, i.e. one network per child.

In both Dirichlet regressions, study population and child age-group were the predictors. These were conducted using the *DirichletReg* package.

Second, establishing differences in the aggregate involvement of the different categories of potential allomother is required to understand allomothering from a systems perspective. This is because it is necessary to incorporate the fact that some categories of helper are more available than others, as discussed above. To test for differences in the aggregate involvement of different categories of potential allomother we first applied a centred log-ratio (clr) transformation to the aggregate network composition data using the *compositions* package. Compositional data is constrained because it consists of multiple proportions which are between 0 and 1, add up to 1 and are non-independent. Clr transformations are frequently used in compositional data analysis to transform the data into a format which is appropriate for the application of standard statistical techniques.

We then conducted pairwise Wilcoxon signed-ranked permutation tests to test for significant difference in the aggregate involvement of (1) different relationship categories; and (2) different life-stage categories of potential allomothers. Permutation tests were chosen since they are better able to handle tied data, they were conducted using the *coin* package. We applied Bonferroni corrections for multiple testing.

We also conducted a Wilcoxon signed-rank permutation test to examine whether the aggregate involvement of all categories of relative combined was significantly greater than that of unrelated campmates. We conducted another Wilcoxon signed-rank permutation test to examine whether the aggregate involvement of reproductively inactive campmates was significantly greater than that of reproductively active campmates. These Wilcoxon signed-rank permutation tests were conducted separately for each population.

Third, we also wished to compare the involvement of an average individual of each category *when they are available*, i.e. alive and co-residing with a child. To examine the factors that predict the involvement of an individual available potential allomother we conducted mixed-effects models on the clr transformed data.

These mixed-effects models were conducted at the dyad level and the dependent variable was the involvement of a given potential allomother in a given child's network. All possible dyads were included, thus each child and each potential allomother appear multiple times in the analysis. Therefore, allomother ID and child ID were included as crossed-random effects. We conducted several of these mixed-effects regressions on the clr transformed data, one for each of the following predictors:

1. Relationship type of the potential allomother-child dyad. This variable could take the following values – fathers; sibling; grandmother; grandfather; aunt/uncle; cousin; unrelated campmate.
2. Coefficient of relatedness of the potential allomother-child dyad. This variable could take the values – most involved unrelated allomother; *r* = 0; *r* = 0.125; *r* = 0.25; *r* = 0.5. In order to compare like for like, we also followed this up with a model adding ‘most involved relative outside of the nuclear family’ as an extra value.
3. Life-stage of the potential allomother. This variable could take the following values – subadult; adult; post-reproductive campmate; most involved subadult; most involved adult; most involved post-reproductive campmate.

The reason for including ‘most involved’ individuals from certain categories in (2) and (3) is because in categories where many individuals are available, their relative involvement is likely to be highly skewed. For instance, the average involvement of an unrelated potential allomother may be low, but the involvement of the most involved unrelated allomother may be very high.

These mixed-effects models were conducted using the package *lmerTest*, and were run separately for the Agta and BaYaka.

Fourth, the initial findings highlighted notable differences in grandmaternal involvement between the two populations. Therefore, we examined whether this may be a product of differences in the availability of grandmothers, as well as differences in the extent to which they were busy with mothering duties. We used chi-square tests to test for differences between the Agta and BaYaka in the proportion of children who had (1) a co-resident grandmother and (2) a co-resident grandmother with no dependent offspring of her own.

## Results

### Agta and BaYaka children have strikingly similar close-proximity networks and rely on the availability of several categories of related and unrelated campmates

**Figure 1.**
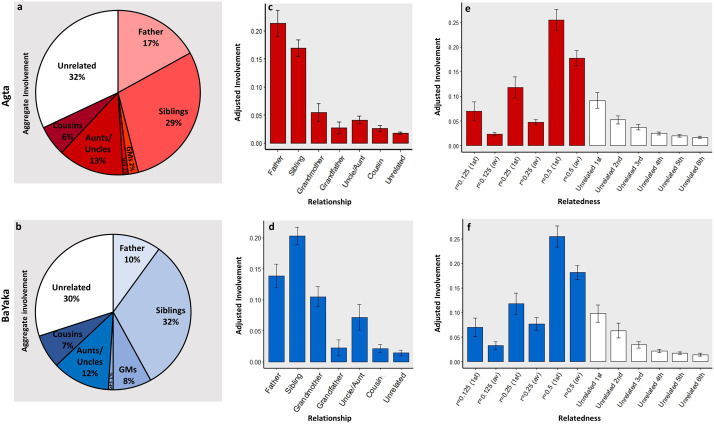
Aggregate and adjusted involvement of different categories of potential allomother. Error bars represent standard errors. The following are calculated by averaging the data from the close-proximity networks of all children in a given population. (a and b) The aggregate involvement of each category of potential allomother (*n* = 46 children (29 Agta)). GMs refers to grandmothers and GFs refers to grandfathers. (c and d) The adjusted involvement of each category of potential allomother (*n* = 46 children [29 Agta]). (e and f) The adjusted involvement of an average relative (av), and the most involved relative (first), with coefficients of relatedness 0.125/0.25/0.5 respectively, and the involvement of the six most involved unrelated campmates (*n* = 47 children (28 Agta)). For example, child *x* has two relatives with whom they share a coefficient of relatedness of *r* = 0.25, who were responsible for 20 and 10% of their close proximity interactions respectively. For this child, the adjusted involvement of *r* = 0.25 (av) would be 15%, and of *r* = 0.25 (first) would be 20%.

Table 2 provides demographic information for the children in each population as well as information on the availability of different categories of potential allomother. The proximity data suggest that allocare may occur extensively in both populations as mothers only account for 22 and 21% of children's close-proximity interactions in the Agta and BaYaka, respectively. The results show that the constitutions of children's close-proximity networks in both populations are very similar from a systems perspective. The aggregate involvement of each category of potential allomother is virtually identical except with respect to grandmothers and fathers, the former having greater aggregate involvement among the BaYaka and the latter among the Agta (Figure 1a and b). Using Dirichlet regression we found that the association between study population and the aggregate involvement of grandmothers approached statistical significance (*p* = 0.051; *n* = 46 (29 Agta)); this was not the case for any other relationship category (see Table S2 for full results). The aggregate involvement of grandmothers in the networks of Agta and BaYaka children was 2 and 8% respectively.

**Table 2.** Focal children demographic characteristics and potential allomother availability

Variable	Agta, *n* = 30	BaYaka, *n* = 19
*Sex*		
Male	20	11
Female	10	8
*Age* (mean ± SD)	1.8 ± 1.1	2.0 ± 1.2
*Network size*^a^ (mean ± SD)	13.9 ± 4.6	11.7 ± 2.9
*Availability*^b^ (mean ± SD)		
Mother	0.97 ± 0.2	1.0 ± 0.0
Father	0.8 ± 0.4	0.8 ± 0.4
Sisters	0.7 ± 1.1	0.9 ± 0.8
Brothers	1.1 ± 1.1	0.8 ± 0.9
Grandmothers	0.4 ± 0.5	0.8 ± 0.5
Grandfathers	0.5 ± 0.5	0.5 ± 0.5
Uncles/aunts	2.8 ± 2.3	1.9 ± 1.3
Cousins	3.4 ± 3.4	2.7 ± 1.9
Unrelated	20.1 ± 7.2	35.1 ± 14.5

a A potential allomother was considered part of a child's close-proximity network if their involvement was greater than 1%.

b Availability refers to the number of potential allomothers in that category that live in the same camp from the perspective of an average child.

In both populations unrelated campmates and siblings stand out, each were responsible for ~30% of the time children spend in close proximity to others. Their combined aggregate involvement in the networks is more than three timese the combined aggregate involvement of fathers and grandmothers. Excluding siblings, unrelated campmates had significantly higher aggregate involvement than each category of relative (*n* = 46 (29 Agta); Figure 1a and b). All of these differences remained significant at the *p* < 0.001 level after applying a Bonferonni correction for conducting multiple pairwise tests. That is not to say that genetic relatedness is unimportant. The aggregate involvement of all categories of relative *combined* comprised approximately 70% of children's close-proximity time in both populations and was significantly higher than unrelated campmates among both the Agta (*p* < 0.001; *n* = 29) and the BaYaka (*p* = 0.007; *n* = 17).

Fathers and grandmothers are the only categories in which the adjusted involvement is greater than aggregate involvement (Figure 1a–d), highlighting that these potential allomothers are not always available to children. For instance, in cases where an Agta grandmother co-resides with a grandchild, she is typically responsible for 5% of her grandchild's time in close proximity to potential allomothers (Figure 1c). However, the aggregate involvement of Agta grandmothers is only 2% because a majority of children do not live with a grandmother (Figure 1a; Table 2).

The adjusted involvement of unrelated campmates is 1.8 and 1.4% in the Agta and BaYaka respectively; in both populations this is the lowest of all relationship categories (Figure 1c and d). Accordingly, in the corresponding mixed-effects model, being an unrelated campmate was associated with significantly lower involvement than being a member of any other relationship category, except in the case of Agta grandfathers where the association approached significance (*p* = 0.054) (Agta: *n* = 866 dyads; BaYaka: *n* = 736 dyads; see Table S3 for full results). Although most unrelated campmates have little involvement, there are typically two or three who are heavily involved in a child's close-proximity network (Figure 1e and f). In both populations, a child's three most involved unrelated campmates are together responsible for ~20% of that child's total time in close proximity to potential allomothers. Furthermore, the most involved unrelated campmate spends more time in close proximity to a child than an *average* relative sharing a coefficient of relatedness of 0.25 (Figure 1e and f). The corresponding result in the mixed-effect models was significant for the Agta (*p* = 0.007) and approached significance for the BaYaka (*p* = 0.095) (Agta: *n* = 866 dyads; BaYaka: *n* = 736 dyads; see Table S4 for full results). That is not to imply that relatives outside of the nuclear family are generally less important. When we compared like for like, the *most* involved relative outside the nuclear family was more involved than the most involved unrelated campmate (Figure 1e and f), although these differences were not statistically significant in either population (Table S5).

We divided the sample into children under 2 years old and children 2 years old and above. At the aggregate level, unrelated campmates and siblings remain the two most involved categories by quite some margin for both age-groups in both populations (Table 3). This margin is even more pronounced in children 2 years old and above. In a Dirichlet regression, child age-group was positively associated with the aggregate involvement of siblings (*p* < 0.001) and unrelated campmates (*p* = 0.059; *n* = 46 (29 Agta)). There were no associations between age-group and any other relationship category which either were significant or approached statistical significance (Table S2).

**Table 3.** Aggregate involvement (%) of potential allomothers by relationship type and child age-group

Population	Age-group	Father	Siblings	Grandmother	Grandfather	Uncle/aunt	Cousins	Unrelated
Agta	< 2 years (*n* = 15)	16.9	24.3	3.0	1.8	17.7	8.7	27.7
	≥ 2 years (*n* = 14)	16.9	33.4	1.4	1.0	7.0	4.0	36.2
BaYaka	< 2 years (*n* = 9)	10.8	28.0	9.6	1.7	16.9	4.4	28.6
	≥ 2 years (*n* = 8)	10.3	35.7	5.7	0.1	5.7	9.9	32.6

#### Variation between the Agta and BaYaka in grandmaternal involvement

As noted above, the study population had a predictive effect on the aggregate involvement of grandmothers which approached statistical significance (Figure 1a and b). This was not the case for any other category of potential allomother; therefore we investigated this difference further. The difference is in part related to availability. When we examined a larger dataset, beyond just those camps where the motes study took place, we found that BaYaka children under the age of 4 were much more likely to have a co-resident grandmother than their Agta counterparts (65.9 vs. 44.7%; *χ*^2^ = 4.6, *p* = 0.033, *n* = 155 (114 Agta)). Furthermore, even when an Agta grandmother is co-residing with a grandchild, her involvement is approximately half that of a BaYaka grandmother (Figure 1c and d). These two trends may be tied to women's fertility schedules. The higher total fertility rate among the Agta (8.07) compared with BaYaka (6.07; Table S6) increases the likelihood that a given Agta child's grandmother resides in a different camp since she is more likely to have other grandchildren living elsewhere. Moreover, there is a later cessation of reproduction among Agta women (Figure S3). Consequently, the likelihood that a co-resident Agta grandmother does not have any dependent offspring of her own, i.e. is not busy being a mother, is much lower than that of a BaYaka grandmother (37.3% vs. 73.1%; *χ*^2^ = 7.5, *p* = 0.006, *n* = 77 (51 Agta)).

### Both reproductively active and inactive helpers are involved in children's close-proximity networks in both populations

**Figure 2.**
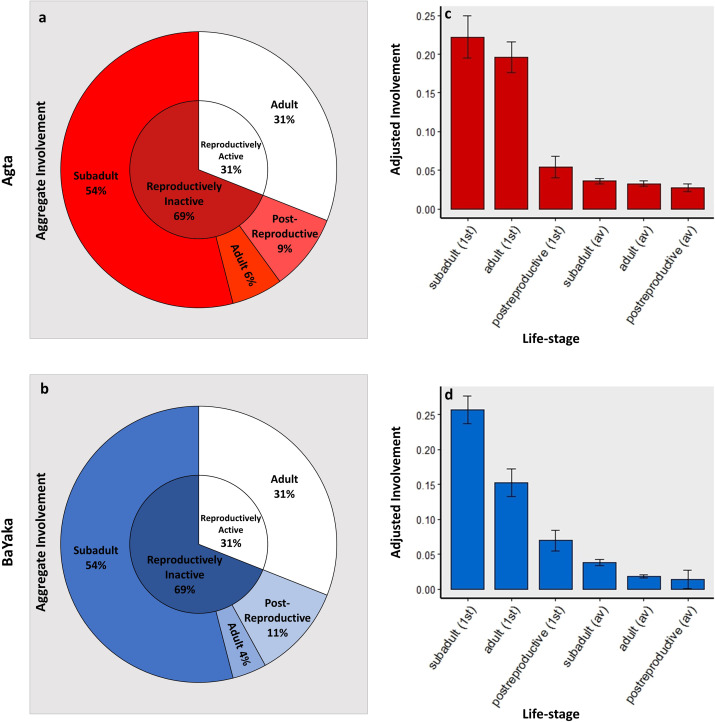
Both reproductively active and inactive camp members are involved in children's close-proximity networks. Results for the Agta in red and the BaYaka in blue. Error bars represent standard errors. See methods for definitions of life-stages and reproductive status. (a and b) The inner-ring is the aggregate involvement of reproductively active and inactive campmates respectively; the outer ring dissects this involvement by life-stage. (*n* = 49 children (30 Agta)). (c and d) The adjusted involvement of children's most involved (first) and average (av) potential allomother of each life-stage (*n* = 49 children (30 Agta)).

Our results also revealed a very strong similarity between the Agta and BaYaka in the involvement of campmates of different life-stages in children's close-proximity networks (Figure 2). Accordingly, in the Dirichlet regression, there was no significant association between study population and the aggregate involvement of subadults, adults or post-reproductive potential allomothers (*n* = 49 (30 Agta); Table S7). In both populations the aggregate involvement of subadults was the highest at 54% and the aggregate involvement of post-reproductive campmates the smallest at 9% and 11% in the Agta and BaYaka, respectively. We conducted Wilcoxon signed-rank permutation tests and found that the aggregate involvement of subadults was significantly higher than adults (*p* < 0.001), and the aggregate involvement of adults was significantly higher than post-reproductive campmates (*p* < 0.001) (*n* = 49 (30 Agta)). The involvement of potential allomothers in all life-stages included both related and unrelated helpers (Table 4).

**Table 4.** Aggregate involvement split by life-stage and relatedness status

	Subadult		Adult		Postreproductive	
Population	Related	Unrelated	Related	Unrelated	Related	Unrelated
Agta	40%	14%	23%	14%	4%	5%
BaYaka	43%	11%	20%	15%	6%	5%

The low aggregate involvement of post-reproductive individuals is not solely a product of low availability since this trend is also reflected in our comparison of the most involved allomother from each life-stage (Figure 2c and d). In the mixed-effects models (Agta: *n* = 890 dyads; BaYaka: *n* = 790 dyads), being the most involved post-reproductive individual was associated with significantly lower involvement than being the most involved subadult (Agta: *p* < 0.001; BaYaka: *p* < 0.001) and adult (Agta: *p* < 0.001; BaYaka: *p* = 0.025; Table S8).

The trends are similar across the networks of children in each age-group (Table 5). In the Dirichlet regression, there was no significant association between child age-group and the aggregate involvement of adults or post-reproductive campmates (*n* = 49 (30 Agta); Table S7). However, there was a positive association between child age-group and the aggregate involvement of subadults which approached statistical significance (*p* = 0.060).

**Table 5.** Aggregate involvement (%) of potential allomothers by life-stage and child age-group

Population	Age-group	Subadult	Adult	Post-reproductive
Agta	< 2 years (*n* = 16)	51.0	38.7	10.2
	≥ 2 years (*n* = 14)	56.5	36.3	7.2
BaYaka	< 2 years (*n* = 10)	51.9	35.0	13.1
	≥ 2 years (*n* = 9)	56.7	34.2	9.1

Overall, in both of the populations, children spent 69% of their close-proximity time with reproductively inactive potential allomothers, significantly more than the 31% with reproductively active campmates (Agta: *p* < 0.001, *n* = 30; BaYaka: *p* < 0.001, *n* = 19; Figure 2a and b). Importantly, we found that 22% of Agta and 16% of BaYaka adults were reproductively inactive. That is to say, they had either (1) not had a live birth in the previous seven adult years or (2) they had not left their natal household *nor* had a live birth. Their aggregate involvement was 6 and 4% in the Agta and BaYaka respectively. This is noteworthy since it is the contributions of these individuals which are being referred to in traditional definitions of cooperative breeding in non-human animals.

### The core involvement of subadults, unrelated campmates and siblings is consistent across hunter–gatherer societies

The Agta and BaYaka close-proximity networks presented here are strikingly similar in terms of core contributions of siblings, unrelated campmates and subadults, whereas the contributions of fathers and grandmothers vary between the populations. We searched for data on hunter–gatherer allomothering in other societies and found very similar trends across ecologies, from savannahs to deserts to tropical forests (Table 6). Siblings (28–32%) and unrelated (30–40%) allomothers are consistently the two highest contributors to childcare. The only exception to this is among the Australian Martu where the author notes a biased sample in which 70% of the sampled children did not have any siblings over the age of 5 (Scelza, 2009). The contribution of subadults is also consistently high across populations where data is available (54–62%).

**Table 6.** Comparison of allocare trends across hunter–gatherer societies

Population^a^	Subadults^b^	Unrelated	Siblings	Father	Grandmothers
Agta	54	32	29	17	2
BaYaka (Mbendjele)	54	30	32	10	8
Efe^c^	56	34	32	10	4
BaYaka (Aka)^d^	NA	NA	30	11	15
Hadza^e^	62	40	28	19	10
Martu^f^	NA	NA	7^g^	3	21
!Kung^h^	NA	NA	NA	17	NA
Mean (Median)	56.5 (55)	34.0 (33.0)	26.3 (29.5)	12.4 (11)	10 (9)

Entries represent aggregate allomaternal contributions provided by each category of allomother.

a Sources cited either present the figures shown here or provide the data required to calculate the figures shown here. Details of how the calculations were conducted can be found in the supplementary material.

b A subadult can also be a sibling or an unrelated campmate, hence row totals can exceed 100%.

c Ivey (2000).

d Helfrecht et al. (2020).

e The Hadza data on the allomaternal contributions of siblings, fathers and grandmothers are from Marlowe (2005b). However, Marlowe (2005b) does not present the data required to calculate the contributions of unrelated and subadult allomothers. Therefore, the figures for unrelated and subadult allomothers are derived from a study that specifically examined which allomothers spent time holding infants and toddlers (Crittenden & Marlowe, 2008).

f Scelza (2009), as summarised in Kramer (2010).

g The author highlights that the low sibling contribution may be an underestimate resulting from features of the specific sample in the study – 40% of the focal children were first-borns and another 30% had no siblings over the age of five.

h Kruger and Konner (2010). This study did not examine all forms of allocare, rather it specifically focused on which allomothers respond to infant crying.

The contribution of fathers to direct care is more variable across societies but never reaches 20%. Grandmaternal contributions are very variable ranging from 2 to 21%. Overall, it appears that substantial involvement of siblings, unrelated campmates and subadults may be the most consistent features of non-maternal direct investment across hunter–gatherers, whereas the aggregate involvement of fathers and grandmothers is lower and more variable than these categories.

## Discussion

Here, we found several striking similarities between the close-proximity networks of Agta and BaYaka children. These are mirrored in available data on allomothering systems of other hunter–gatherer populations living across vastly differing ecologies, from savannahs to deserts to tropical rainforests.

Unrelated campmates, siblings and subadults were the most involved in children's close-proximity networks at the aggregate level. The involvement of fathers and grandmothers was the most variable between the two populations. Importantly, both related and unrelated and reproductively active and inactive campmates spend substantial amounts of time in close-proximity with infants and toddlers. This, in and of itself, suggests that a label of cooperative breeding *or* communal breeding does not encapsulate hunter–gatherer allomothering at a systems level.

The differences between the involvement of categories at the aggregate vs. adjusted level highlight an association between helper availability and allomothering systems. Below we discuss the involvement of different categories of potential allomother, and where relevant, the forces that shape their availability and involvement. Accordingly, we are able to consider similarities and differences with descriptions of cooperative and communal breeding in other taxa.

### Reproductively inactive potential allomothers

Reproductively inactive campmates were responsible for approximately 70% of children's time in close-proximity to potential allomothers. Their involvement satisfies loose definitions of cooperative breeding, and in some cases has parallels with traditional definitions.

#### Grandmothers and post-reproductive campmates

One of the reasons it may have become common to describe humans as cooperative breeders is due to the heavy emphasis on post-reproductive grandmothers (Hawkes & Coxworth, 2013). They are reproductively inactive helpers; and the extended post-reproductive lifespan evolved via reallocation of energy away from reproduction towards somatic effort to increase vigour and buffer mortality risks in later life (Hawkes, 2003). Thus, the emergence of grandmothering involved a reduction in personal reproductive effort, which was favoured because of the inclusive fitness benefits of helping a closely related reproductively active female.

Regardless of these similarities, we found that the involvement of grandmothers and other post-reproductive campmates to be modest, particularly among the Agta. The low aggregate involvement appears to be partially due to low availability, presumably because of mortality and multi-local residence. The low adjusted involvement of an Agta grandmother may be due to women's late cessation of reproduction, resulting in grandmothers being busy providing care to their own offspring. Correspondingly, Ache women stop reproducing even later in life (Migliano et al., 2007), and anthropologists working with the Ache emphasise that grandmothers are unimportant (Hill & Hurtado, 2009). In contrast, Scelza (2009) highlights that Martu grandmothers can make vital allomaternal contributions because most of them have finished their personal reproduction and are no longer busy rearing their own children. Indeed, the involvement of grandmothers is one of the most variable features of allomaternal systems across hunter–gatherer societies (Table 6); we suspect that this may be driven by variability in fertility schedules.

We emphasise that Table 6 and the motes data only examine grandmaternal involvement in childcare. We have not assessed the contributions grandmothers make in the domains of provisioning and knowledge sharing, which have both been highlighted by other anthropologists (Hawkes et al., 1997; Scelza & Hinde, 2019). Additionally, the data presented is from the perspective of an average *individual* child. As such, we are not attempting to evaluate the inclusive fitness gains of a post-reproductive lifespan. This would require taking the *grandmother's* perspective and examining her allomaternal contributions across all domains to all her grandchildren.

#### Reproductively inactive adults

One of the primary motivations for studying traditional cooperative breeding in non-human animals is to understand why sexually mature helpers would remain non-reproductive and help raise the offspring of another adult (Hatchwell, 2009; Lukas & Clutton-Brock, 2012b). As far as we are aware, among hunter–gatherers there has been only one quantification of the allomaternal contribution of reproductively inactive adults who are of reproductive age. Strikingly, 32% of reproductive-aged Efe women were reproductively inactive; however, they only provided 12% of allocare (Ivey, 2000). According to our definition, we found that 22% of Agta, and 16% of BaYaka, reproductive-aged adults were reproductively inactive. Their aggregate involvement was approximately 5% in both populations. Thus, a majority of Agta and BaYaka allomothering does not fit traditional definitions of cooperative breeding.

#### Siblings

Siblings, alongside unrelated campmates, had the highest involvement in the close-proximity networks of infants and toddlers, and their aggregate contribution is high among other foraging societies (Table 6). The high-relatedness between siblings arises owing to pair-bonding in both humans and cooperatively breeding non-human animals. In many cases, such as white fronted bee eaters, the reason for delayed dispersal of sibling helpers is low resource availability (Emlen, 1982). Hunter–gatherer siblings are not ‘delaying’ dispersal *per se*; they remain sexually immature when many of their younger siblings are being born. However, it is still difficulties in resource acquisition – arising from a high skill foraging niche rather than low resource availability – which drove the evolution of an extended immature period, and in turn makes siblings available as helpers. That is to say, it is an ecological constraint that favours helping behaviour over commencing personal reproduction. In this sense, the vital contribution of siblings has some similarities to cooperative breeding in non-human animals.

#### Subadults (related and unrelated)

Subadults had greater aggregate involvement than adults and post-reproductive campmates in the proximity networks described here, and we found this trend to be mirrored in studies of childcare across hunter–gatherer populations (Table 6). They are particularly interesting when considering the evolution of allomothering systems as they are both recipients and providers of allomothering. They remain nutritionally dependent in the natal household for an extended period while they acquire the necessary subsistence skills. As such, mothers rely on allomaternal support to raise multiple dependent offspring simultaneously. Similarly, complex foraging niches and the need for skill acquisition can lead to extended dependence and a reliance on allomothering in other species such as white-winged choughs (Heinsohn, 1991), and greater dependency loads are associated with the evolution of allomothering more generally (Lukas & Clutton-Brock, 2012b). While hunter–gatherer subadults create a need for allomaternal provisioning as observed in other species, the results and inter-population comparison presented here highlight that they also make the largest allomaternal contribution to childcare.

Subadult care can play an important role in freeing mothers to engage in other tasks; this can happen even if mothers are present. For instance, BaYaka subadults that attend to infants on foraging expeditions increase the productivity of nursing mothers (Jang et al., 2022). As infants become less dependent on their mother's milk, the potential for subadults to release mothers from caretaking duties increases further. Across foraging societies, older infants begin spending much of the day in mixed-age playgroups where they are supervised by older children (Lew-Levy et al., 2017; Page et al., 2021; Salali et al., 2019). Correspondingly, we found that subadults and siblings spent more time with those infants/toddlers who were 2 years old and above.

It is important to highlight that the motivation for subadult allomothers to spend time in playgroups is not primarily to provide allocare. Key proximate motivations are probably the enjoyment of play, spending time with friends and being active. Play also has important ultimate functions unrelated to allocare, such as the acquisition and transmission of subsistence skills and social norms (Lew-Levy et al., 2017, 2018). Although allomothering is not the driving force behind subadult participation in playgroups, they are still the ones supervising and providing care to any infants and toddlers who are present. Correspondingly, we previously found that as Agta infants and toddlers spend more time in playgroups, their mothers devote less time to childcare and are freer to engage in other tasks (Page et al., 2021).

This supervision and care that occur in these playgroups blend features of cooperative and communal breeding, including reproductively inactive helpers, some of whom are unrelated. When infants and toddlers are pooled in space, then mothers can engage in other activities while other caregivers supervise the playgroup. This is a similar to the crèches of communally breeding mammals. For example, among free-living house mice and black-and-white ruffed lemurs, crèching decreases the time mothers spend at the nest and increases their foraging time (Auclair et al., 2014; Baden et al., 2013). However, in contrast to communal breeding systems where reproductively active mothers take turns in supervising the crèche, in hunter–gatherer playgroups it is reproductively inactive subadults who supervise. Their availability to do so is a result of the late age of maturity already discussed, which is more similar to a cooperative breeding system. Equally, subadults in the same playgroup come from numerous reproductively active households and are often unrelated. Therefore, the processes that lead to the high availability and involvement of subadults are a combination of those that set the stage of cooperative and communal breeding.

### Reproductively active helpers

#### Fathers

Pair-bonding provides a foundation for cooperative breeding in birds and mammals as it produces high relatedness between a female's offspring (Cornwallis et al., 2010; Lukas & Clutton-Brock, 2013). In our sample 80% of Agta and BaYaka children had access to a co-resident father. Although fathers are reproductively active, their contribution is not a form of communal breeding since they are providing care to their own offspring. Bi-parental care is normative among cooperatively breeding birds, and common in cooperatively breeding mammals (Griesser et al., 2017; Opie et al., 2013).

We found that the time fathers and grandmothers spend in close proximity to children is relatively similar among the BaYaka. However, among the Agta, the relatively higher involvement of fathers may be a response to the lower involvement and availability of grandmothers. This may be because the payoff to paternal care increases when their offspring are lacking grandmaternal support. Indeed, there is an inverse relationship between the paternal and grandmaternal investment across Aka camps (Meehan, 2005), and among the Martu, the substantial care provided by grandmothers is speculated to underpin the low paternal effort (Scelza, 2009).

#### Unrelated campmates

We found unrelated campmates to have the largest aggregate involvement alongside siblings in both the Agta and BaYaka; this is the case in other hunter–gatherer societies where their contribution has been calculated (Table 6). About half of this involvement was of reproductively active adults. Their availability is a result of bilocal residence patterns which results in low relatedness between households in a camp (Dyble et al., 2015). This is distinct from cooperative breeding, which evolves in the context of high relatedness (Lukas and Clutton-Brock, 2012a).

Using the motes, we previously found that Agta allomothering of unrelated children is best predicted by reciprocity (Page et al., 2019). Thus, the involvement of unrelated reproductively active adults can be effectively characterised as communal breeding – multiple reproductive pairs sharing caregiving responsibilities with one another. This is arguably the only contribution that fits neatly within the existing classification schemes.

### Limitations

The primary limitation of this study is that the motes do not provide information on what is occurring when a potential allomother is in close proximity to a child. Certain categories of allomother may be more likely to be providing high investment care, as observed among Martu Grandmothers (Scelza, 2009). Additionally, some categories of helper may be more skilled at providing care. It has been suggested elsewhere that subadult allomothers may be ‘learning to mother’ (Crittenden & Marlowe, 2008; Fairbanks, 1989); therefore, it may take time before their care will match the quality of care of older caregivers. Equally, some allomothers may be more likely to spend time in close proximity to a child when no other potential allomothers are around. For all these reasons, a potential allomother's involvement in a child's close-proximity network cannot be assumed to be equivalent to the *value* of their allomaternal contribution. Despite these limitations, we can consider close proximity as the minimum form of, and minimum requirement for, allocare. Therefore, the results here still provide insight into the floor and ceiling of the contributions to childcare provided by each category of allomother. The fact that our results are mirrored in childcare studies measuring other caregiving behaviours provides support for their validity.

The contributions allomothers make via provisioning may follow different trends to those presented here. In particular, provisioning systems do not rely so heavily on subadults and siblings, who have been shown to remain net consumers of calories for an extended period in other hunter–gatherer societies (Kaplan et al., 2000), although their contribution to household provisioning should not be undervalued since they may target different foods to adults, thus increasing dietary breadth (Crittenden et al., 2013; Kramer, 2014). Equally, the importance of fathers and grandmothers in food provisioning is probably higher than their involvement in close-proximity networks. In fact, there may be some inverse relationship since when fathers are hunting they cannot also be in close proximity to their infants. Provisioning contributions are also likely more variable across ecologies given that the sex division of labour and subadult foraging activities are determined by diverse dietary niches (Kramer, 2005; Marlowe, 2005a).

Finally, accurately valuing the contributions of different allomothers should ideally involve analysing the effect of their help on the fitness of the children receiving the care and their mothers.

Given these limitations, our results do not offer a full valuation of the allomaternal contributions of different helpers and cannot be considered to be a complete description of the allomothering system.

## Conclusion

We lived as hunter–gatherers for the majority of our evolutionary history, thus studying contemporary hunter–gatherers is particularly informative for our understanding of the evolution of human allomothering. Humans are frequently described as cooperative breeders. However, our results highlight that allomothering is widely distributed; it involves reproductively active and inactive, and related and unrelated, helpers.

The forces shaping the availability and involvement of the helpers who are most involved have some similarities to those operating in non-human cooperative and communal breeding systems. However, with the exception of help from reproductively active adults outside of the household, which resembles communal breeding, none of the key contributions fit neatly into existing classification schemes. Moreover, traditional definitions of cooperative breeding focus on help provided by sexually mature individuals who are reproductively inactive; among both the Agta and BaYaka, the involvement of these individuals is very limited. If the purpose of inter-specific classification schemes is to identify similarities across taxa and investigate potentially overlapping evolutionary processes, then it is unhelpful to classify hunter–gatherer allomothering as cooperative or communal breeding. It has some similarities with both systems but also has several features found in neither.

## Supporting information

Chaudhary et al. supplementary material Chaudhary et al. supplementary material

## Supplementary material

The supplementary material for this article can be found at https://doi.org/10.1017/ehs.2024.1.

## Research transparency and reproducibility

Processed data that support the findings of this study are available here: https://osf.io/n4b9e/
