# Supplementary material for: Hunter–Gatherer children's close-proximity networks: Similarities and differences with cooperative and communal breeding systems
Source: Evol Hum Sci. 2024 Jan 31;6:e11. doi: 10.1017/ehs.2024.1 (PMC10955362; doi:10.1017/ehs.2024.1)
Supplement: Chaudhary et al. supplementary material [file S2513843X2400001Xsup001.docx]

**Supplementary Material for:**

**Children’s Close-Proximity Networks among Agta and BaYaka Hunter-Gatherers: Similarities and differences with cooperative and communal breeding systems.**

Nikhil Chaudhary*, Abigail E. Page, Gul Deniz Salali, Mark Dyble, Andrea B. Migliano, Lucio Vinicius, Daniel Smith, James Thompson & Sylvain Viguier

*corresponding author: Nikhil Chaudhary

Email: nc542@cam.ac.uk

**This file includes:**

1. A verification of the motes data using observational focal follows (including Table SV1 and SV2).

2. Key results derived from setting the Agta motes threshold to 1.5 metres (Figures S1 and S2).

3. A worked example of the calculation of aggregate and adjusted involvement (Table S1)

4. Results from Dirichlet and mixed-effects models related to relationship and relatedness categories (Tables S2-S5)

5. Calculation of the total fertility rate for both populations (Table S6). Age-specific fertility schedule in both populations (Figure S3).

6. Results from Dirichlet and mixed-effects models related to allomother life-stage (Tables S7 & S8)

7. Information on calculations of the allocare trends across hunter-gatherer societies which are presented in Table 6 of the main-text.

**1. Verifying the motes**

In this study focusing on the role of close proximity in allocare systems it is necessary to confirm the motes data being recorded are actually reflecting something meaningful, i.e. spending time in close enough proximity to facilitate supervision and intervention if required. We did so by comparing the data with data derived from observational focal follows of a smaller sample of children.

A focal child was observed for 12 hours, split into three segments: morning (6am-10am); afternoon (10am-2pm); evening (2pm-6pm), each observation segment was conducted on a different day in an attempt to minimise biases. During each observation hour, notes were taken every 30 seconds for the first 45 minutes followed by a break for 15 minutes. During each 30 second interval the focal child was observed for 20 seconds, and then details were noted by the researcher for the next 10 seconds. Amongst other things, we recorded whether an individual was in proximity to a child. An individual would only be recorded as proximate if they were within 3 metres from the focal child and either providing some form of high-investment care already (e.g. feeding or soothing) or in a position where they could supervise the child and intervene if required. Thus, recordings of proximity reflected a form of proximity that is meaningful for caregiving.

In Page et al. (2019) we validated the motes data as a proxy for allocare by comparing motes data for five children with data derived from observational focal follows that were conducted on the exact same days. This comparison is presented in Table S1. We do not have the same data available for the BaYaka because we did not observe any children on the exact days that they were wearing the motes. However, nine of the children in our motes sample were observed, but on different days to the motes data collection. In Table S2 we calculate the relative involvement of different categories of potential allomothers based on proximity data from the motes and observations respectively.

The overall picture of proximity networks that emerges from both methods is similar. To examine whether the motes were capturing similar events to the observations, we calculated a weighted change from the observation results to motes result. For instance, the motes produce an estimate of BaYaka sibling involvement which is 11.1% lower [(27-24)/27] than the observation estimate (Table S2). Siblings comprise 27% of proximity time according to the observations, so the weighted difference for siblings is 3% [11.1*0.27]. We then sum the weighted difference across all categories of allomother to produce an overall weighted change between the motes and observations. This weighted change is 14% in the Agta data and 16% in the BaYaka data (it is noteworthy that it is the motes which are not subject to human error). This level of discrepency is comparable to inter-observer reliability of measurements of proximity in observational studies e.g. 80-90% (Meehan et al., 2013), and 82% (Hewlett et al., 1998). This suggests that the motes were capturing a type of close proximity that is meaningful for understanding allomothering systems.

**Table SV1: Verification of the motes among the Agta taken from Page *et al.* 2019.** This is a comparison of motes and observational data recording the mean (SD) proportion of time different categories of caregivers spent in proximity with five focal children. Data were collected on the same days.

|  | Mother | Siblings | Unrelated | Father | Grandparents | Other kin |
| --- | --- | --- | --- | --- | --- | --- |
| Observations | 37 (26) | 24 (19) | 24 (20) | 19 (19) | 2 (1) | 7 (8) |
| Motes | 34 (26) | 24 (13) | 23 (13) | 11 (5) | 6 (6) | 7 (7) |

**Table SV2: Comparison of motes and observational data recording the mean (SD) percentage of a child’s proximity time which was spent with different categories of caregiver among the BaYaka**. Data are based on nine of the children from the motes sample who were also observed, but on different days.

|  | Mother | Siblings | Unrelated | Father | Grandparents | Other kin |
| --- | --- | --- | --- | --- | --- | --- |
| Observations | 25 (13) | 27 (18) | 23 (16) | 8 (6) | 8 (8) | 9 (7) |
| Motes | 21 (6) | 24 (20) | 26 (20) | 8 (7) | 7 (5) | 14 (12) |

**2. Agta motes results with motes threshold of 1.5m**

**Figure S1: Involvement of different categories of potential allomother among the Agta with a motes threshold of 1.5m.** a) The aggregate involvement of each category of potential allomother in children’s close proximity networks (n=23). GMs refers to grandmothers and GFs refers to grandfathers. b) The adjusted involvement of each category of potential allomother (n=23). c) The adjusted involvement of an average relative (av), and the most involved relative (1^st^), with coefficients of relatedness 0.125/0.25/0.5 respectively; and the per capita involvement of the six most involved unrelated campmates (n=23). For example, child x has two relatives with whom they share a coefficient of relatedness of r=0.25, who were responsible for 20% and 10% of their close proximity interactions respectively. In this case, the adjusted involvement of r=0.25 (av) would be 15%, and of r=0.25 (1^st^) would be 20%.


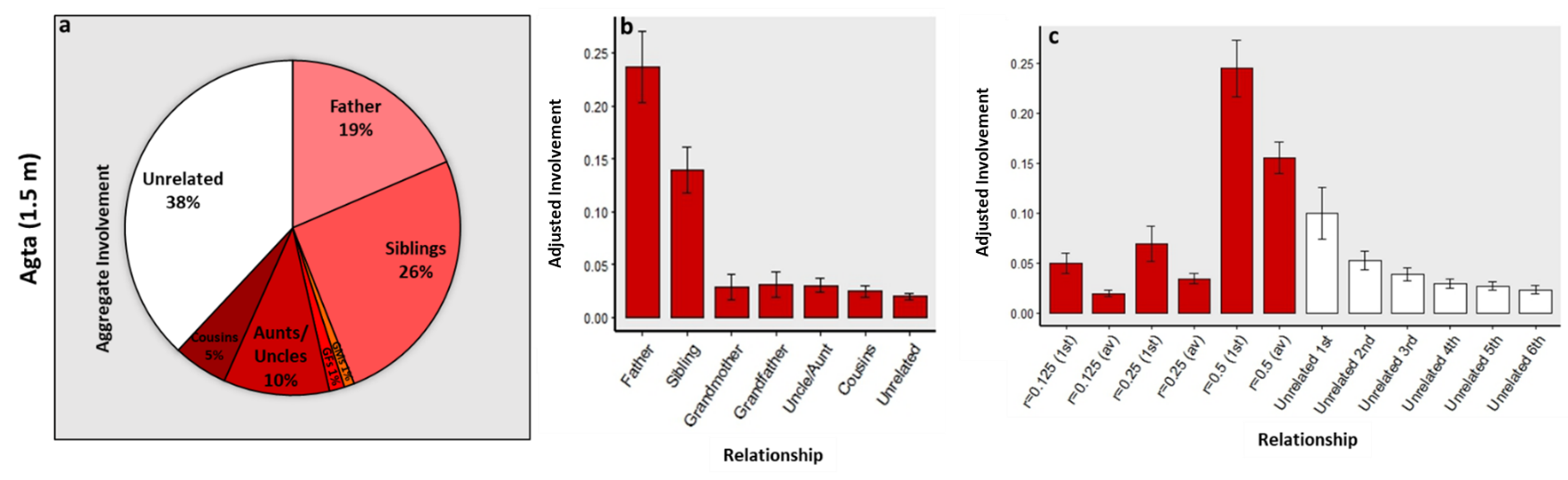


**Figure S2: Both reproductively active and inactive camp members are involved in children’s close proximity networks.** Error bars represent standard errors. Adults are individuals of reproductive age, and reproductively inactive adults are those who either i) have not had a live birth in over seven years or ii) have not left their natal household nor had any children. a) Inner-ring is the aggregate involvement of reproductively active and inactive campmates respectively; outer ring dissects this involvement by life-stage (n=23). b) The adjusted involvement of children’s most involved (1^st^) and average (av) potential allomother of each life-stage (n=23).


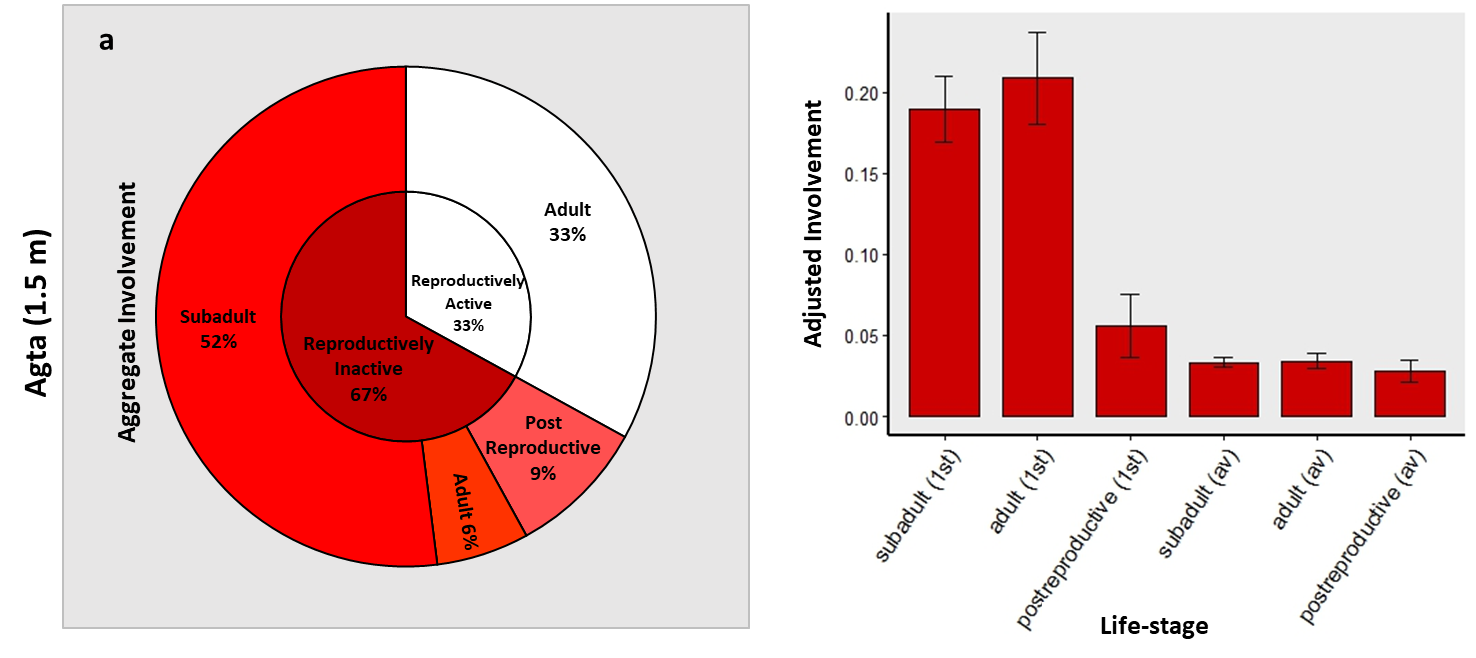


**3. Worked example of key measures**

In hypothetical Population A children are only ever in close proximity with their nuclear family so fathers and siblings are the only categories of potential allomother. Child 1 has a father and 3 siblings, and Child 2 has no father and has 2 siblings. ‘Involvement’ is the proportion of time a child spent in close proximity with a given potential allomother as a proportion of the total time they spent in close proximity to all potential allomothers. Below we show how aggregate and adjusted involvement are calculated from involvement.

**Table S1: Worked example**

|  | *Involvement* | | | | *Aggregate Involvement* | | *Adjusted Involvement* | |
| --- | --- | --- | --- | --- | --- | --- | --- | --- |
|  | Father | Sibling 1 | Sibling 2 | Sibling 3 | Father | Siblings | Father | Sibling |
| Child 1 | 10% | 55% | 35% | 0% | 10% | 90% [55%+35%+0%] | 10% [10%/1] | 30% [(55%+35%+0%)/3] |
| Child 2 | NA | 60% | 40% | NA | 0% | 100% [60%+40%] | NA | 50% [(60%+40%)/2] |
| Population A | - | - | - | - | 5% [(10%+0%)/2] | 95% [(90%+100%)/2] | 10% [10%/1] | 40% [(30%+50%0/2] |

**4. Results on the effects relationship type and relatedness**

| **Table S2: Results from Dirichlet regression testing effect of age-group and study population on the aggregate composition of networks. The outcome in a Dirichlet model is compositional, i.e. made up of several components of a larger structure.** | | | | |
| --- | --- | --- | --- | --- |
|  |  |  |  |  |
| **Component (n=46 networks [29 Agta])** | **Predictor** | **Coeff.** | **S.E.** | **p-value** |
| Father |  |  |  |  |
|  | Age-Group (< 2 years) | -0.25 | 0.27 | 0.362 |
|  | Population (BaYaka) | -0.14 | 0.28 | 0.626 |
| Siblings |  |  |  |  |
|  | Age-Group (< 2 years) | -1.08 | 0.27 | <0.001 |
|  | Population (BaYaka) | 0.10 | 0.27 | 0.711 |
| Grandmothers |  |  |  |  |
|  | Age-Group (< 2 years) | 0.06 | 0.28 | 0.845 |
|  | Population (BaYaka) | 0.57 | 0.29 | 0.051 |
| Grandfathers |  |  |  |  |
|  | Age-Group (< 2 years) | 0.00 | 0.29 | 0.996 |
|  | Population (BaYaka) | -0.03 | 0.30 | 0.918 |
| Uncle/Aunts |  |  |  |  |
|  | Age-Group (< 2 years) | 0.40 | 0.28 | 0.151 |
|  | Population (BaYaka) | 0.20 | 0.29 | 0.484 |
| Cousins |  |  |  |  |
|  | Age-Group (< 2 years) | 0.11 | 0.28 | 0.694 |
|  | Population (BaYaka) | -0.11 | 0.29 | 0.715 |
| Unrelated campmates |  |  |  |  |
|  | Age-Group (< 2 years) | -0.48 | 0.26 | 0.059 |
|  | Population (BaYaka) | 0.07 | 0.26 | 0.797 |

Results in Tables S3-S5 are from models run on centred log-ratio transformed data, and so coefficients are not intuitive to interpret. The summary statistics in the main-text offer a clearer sense of the magnitude of differences between the involvement of individuals from different categories.

**Table S3: Results from mixed-effects model testing effect of relationship type on allomother involvement.**

|  | **Agta (n=866 dyads)** | | | **BaYaka (n=736 dyads)** | | |
| --- | --- | --- | --- | --- | --- | --- |
| Reference = Unrelated | **Coeff.** | **S.E.** | **p-value** | **Coeff.** | **S.E.** | **p-value** |
| Sibling | 2.43 | 0.16 | <0.001 | 3.45 | 0.20 | <0.001 |
| Cousin | 0.33 | 0.13 | 0.010 | 0.62 | 0.18 | 0.001 |
| Father | 2.98 | 0.25 | <0.001 | 3.36 | 0.30 | <0.001 |
| Grandfather | 0.65 | 0.34 | 0.054 | 2.00 | 0.42 | <0.001 |
| Grandmother | 1.55 | 0.36 | <0.001 | 3.12 | 0.30 | <0.001 |
| Uncle/Aunt | 1.20 | 0.14 | <0.001 | 1.47 | 0.20 | <0.001 |

**Table S4: Results from mixed-effects model testing effect of coefficient of relatedness on allomother involvement.**

|  | **Agta (n=866 dyads)** | | | **BaYaka (n=736 dyads)** | | |
| --- | --- | --- | --- | --- | --- | --- |
| Reference = Unrelated (1st) | **Coeff.** | **S.E.** | **p-value** | **Coeff.** | **S.E.** | **p-value** |
| r=0 | -1.99 | 0.20 | <0.001 | -2.68 | 0.26 | <0.001 |
| r=0.125 | -1.49 | 0.23 | <0.001 | -1.92 | 0.30 | <0.001 |
| r=0.25 | -0.62 | 0.23 | 0.007 | -0.51 | 0.31 | 0.095 |
| r=0.5 | 0.76 | 0.24 | 0.001 | 0.79 | 0.30 | 0.008 |

**Table S5: Results from mixed-effects model testing effect of coefficient of relatedness on allomother involvement (including most involved relative outside of household).**

|  | **Agta (n=866 dyads)** | | | **BaYaka (n=736 dyads)** | | |
| --- | --- | --- | --- | --- | --- | --- |
| Reference = Unrelated (1st) | **Coeff.** | **S.E.** | **p-value** | **Coeff.** | **S.E.** | **p-value** |
| r=0 | -2.01 | 0.21 | <0.001 | -2.69 | 0.26 | <0.001 |
| r=0.125 | -1.55 | 0.24 | <0.001 | -2.07 | 0.31 | <0.001 |
| r=0.25 | -0.84 | 0.24 | 0.001 | -0.85 | 0.32 | 0.007 |
| r=0.5 | 0.89 | 0.25 | <0.001 | 0.74 | 0.29 | 0.013 |
| 0.125≤r<0.5 (1st) | 0.07 | 0.30 | 0.813 | 0.59 | 0.36 | 0.098 |

**5. Table S6: Calculation of total fertility rate.**

|  |  | **Agta** |  |  | **BaYaka** |  |
| --- | --- | --- | --- | --- | --- | --- |
| **Age-Group** | **n** | **Live Births** | **Rate** | **n** | **Live Births** | **Rate** |
| 10-14 | 59 | 0 | 0 | 19 | 0 | 0 |
| 15-19 | 44 | 3 | 0.068182 | 16 | 2 | 0.125 |
| 20-24 | 46 | 17 | 0.369565 | 11 | 4 | 0.363636 |
| 25-29 | 20 | 6 | 0.3 | 12 | 3 | 0.25 |
| 30-34 | 19 | 7 | 0.368421 | 8 | 2 | 0.25 |
| 35-39 | 17 | 5 | 0.294118 | 8 | 1 | 0.125 |
| 40-44 | 14 | 3 | 0.214286 | 10 | 1 | 0.1 |
| 45-49 | 13 | 0 | 0 | 7 | 0 | 0 |
| Sum Rate |  |  | 1.614571 |  |  | 1.213636 |
| TFR ([sum rate]*5) |  |  | 8.072857 |  |  | 6.068182 |

**Figure S3. Age-Specific Fertility Curves for the Agta and BaYaka. Produced using data from Table S3.**

**6. Results on the effects allomother life-stage**

| **Table S7: Results from Dirichlet regression testing effect of age-group and study population on the aggregate composition of networks. The outcome in a Dirichlet model is a compositional, i.e. made up of several components of a larger structure.** |
| --- |

| **Component (n=49 networks [30 Agta])** | **Predictor** | **Coeff.** | **S.E.** | **p-value** |
| --- | --- | --- | --- | --- |
| Post-reproductive campmates |  |  |  |  |
|  | Age-Group (< 2 years) | -0.26 | 0.29 | 0.374 |
|  | Population (BaYaka) | 0.28 | 0.30 | 0.359 |
| Subadults |  |  |  |  |
|  | Age-Group (< 2 years) | -0.59 | 0.31 | 0.060 |
|  | Population (BaYaka) | 0.00 | 0.32 | 0.992 |
| Adult |  |  |  |  |
|  | Age-Group (< 2 years) | -0.46 | 0.31 | 0.140 |
|  | Population (BaYaka) | -0.09 | 0.32 | 0.777 |

**Table S8: Results from mixed-effects model testing effect of allomother life-stage on involvement.**

This model was run on clr transformed data.

|  | **Agta (n=890 dyads)** | | | **BaYaka (n=790 dyads)** | | |
| --- | --- | --- | --- | --- | --- | --- |
| Reference = Post-reproductive (1st) | **Coeff.** | **S.E.** | **p-value** | **Coeff.** | **S.E.** | **p-value** |
| Adult (1st) | 1.84 | 0.34 | <0.001 | 0.94 | 0.42 | 0.025 |
| Subadult (1st) | 1.94 | 0.34 | <0.001 | 1.77 | 0.42 | <0.001 |
| Post-reproductive | -1.34 | 0.28 | <0.001 | -2.46 | 0.29 | <0.001 |
| Adult | -1.11 | 0.27 | <0.001 | -2.13 | 0.33 | <0.001 |
| Subadult | -0.77 | 0.27 | 0.005 | -1.72 | 0.33 | <0.001 |

**7. Calculation of allocare trends presented in Table 6 of main-text.**

*Agta and BaYaka:* The data presented are the results from the current study.

*Efe (Ivey 2000):*

-The value for subadults (56%) is the sum of the mean proportion of allocare provided by nonkin male (4.22%), nonkin female (6.96%), kin male (26.79%), kin female (18.14%) children as presented in Figure 5.

-The value for unrelated campmates (34%) is the sum of the mean proportion of allocare provided by nonkin male children (4.22%), nonkin female children (6.96%), nonkin male adults (2.95%), and nonkin female adults (19.41%) as presented in Figure 5.

-The values for siblings, father, and grandmothers are calculated based on the data in Table 5. Multiplying the mean time caring for an infant of each category of allomother by the number of individuals in each category produces a total of 7065 minutes of allocare, of which 2242 mins (32%) is from siblings, 700 minutes (10%) is from fathers, and 255 mins (4%) is from grandmothers.

*Aka (Helfrecht et al., 2020):*

-The values for father, siblings and grandmothers are calculated based on the data in Table 3 of the supplementary information. For each category of allomother we used the frequency of allomaternal care data to calculate the amount of allocare provided by that category of allomother as a proportion of the total amount of allocare provided by all categories of allomother. We did this for each age-group and then averaged the results across age-groups.

For example, for fathers:

| Age-group (months) | <6 | 6-<12 | 12-<24 | 24-<36 | 36-<48 |
| --- | --- | --- | --- | --- | --- |
| Freq father allocare | 24.11 | 11.82 | 34.45 | 56.77 | 13.33 |
| Freq all allocare | 340.32 | 197.56 | 288.05 | 233.15 | 210.16 |
| Proportion father | 0.07 | 0.06 | 0.12 | 0.24 | 0.06 |
|  |  |  |  |  |  |
| Average proportion | 0.11 |  |  |  |  |

Note row 3 in the table above (freq all allocare) is simply the sum of the frequency of allocare by all categories of allomother, i.e. the column totals of the original table excluding maternal care.

*Hadza (Marlowe, 2005b; Crittenden & Marlowe, 2008):*

-The values for father, siblings and grandmother are calculated using the data in Table 8.1 of Marlowe 2005b. We used the ‘Total’ row for children under four years old. The sum of the percentages of time spent interacting with all categories of allomother is 93.61. The contribution of category x is then calculated by diving the % of time interacting with x by 93.61. For example, for fathers this is 17.37/96.31 = 19%

-The value for unrelated allomothers is calculated based on data on allomaternal holding in Critten & Marlowe, 2008 as presented in Table 1. 12.4% of all holding is by unrelated allomothers and 68.7% of all holding is by the mother. Therefore, the allomaternal contribution of unrelated allomothers is 12.4/(100-68.7) = 40%.

-The 62% subadult contribution reflects the in-text reporting (page 6) that 62% of allomothers were subadults. This is the only approximation we could find of subadult contributions to Hadza allocare.

*Martu (Scelza, 2009 as reported in Kramer, 2010):*

In Table 1 of Kramer, 2010, the proportion of total care Martu children receive from the mother is 32.2%, therefore the proportion of total care which is allocare is 67.8%. Fathers provide 1.7% of total care, which is equivalent to 3% of allocare (1.7/67.8); siblings provide 5% of total care, which is equivalent to 7% of allocare (5/67.8); grandmothers provide 14.3% of total care, which is equivalent to 21% of allocare (14.3/67.8).

*!Kung (Kurger & Konner, 2010):*

P321 reports that 17% of all allomaternal responses to crying were provided by fathers.

**REFERENCES**

Crittenden, A. N., & Marlowe, F. W. (2008). Allomaternal care among the Hadza of Tanzania. *Human Nature*, *19*(3). https://doi.org/10.1007/s12110-008-9043-3

Helfrecht, C., Roulette, J. W., Lane, A., Sintayehu, B., & Meehan, C. L. (2020). Life history and socioecology of infancy. *American Journal of Physical Anthropology*, *173*(4), 619–629. https://doi.org/10.1002/AJPA.24145

Hewlett, B. S., Lamb, M. E., Shannon, D., Leyendecker, B., & Schölmerich, A. (1998). Culture and early infancy among central African foragers and farmers. *Developmental Psychology*, *34*(4), 653–661. https://doi.org/10.1037/0012-1649.34.4.653

Ivey, P. K. (2000). Cooperative reproduction in Ituri forest Hunter-Gatherers: Who cares for Efe infants? *Current Anthropology*, *41*(5). https://doi.org/10.1086/317414

Kramer, K. L. (2010). Cooperative breeding and its significance to the demographic success of humans. *Annual Review of Anthropology*, *39*. https://doi.org/10.1146/annurev.anthro.012809.105054

Kruger, A. C., & Konner, M. (2010). Who Responds to Crying?: Maternal Care and Allocare among the !Kung. *Human Nature*, *21*(3), 309–329. https://doi.org/10.1007/s12110-010-9095-z

Marlowe, F. W. (2005b). Who Tends Hadza Children? In B. S. Hewlett & M. E. Lamb (Eds.), *Hunter-Gatherer Childhoods* (pp. 177–190). Aldine Transaction. https://doi.org/10.4324/9780203789445-13

Meehan, C. L., Quinlan, R., & Malcom, C. D. (2013). Cooperative breeding and maternal energy expenditure among aka foragers. *American Journal of Human Biology*, *25*(1). https://doi.org/10.1002/ajhb.22336

Page, A. E., Thomas, M. G., Smith, D., Dyble, M., Viguier, S., Chaudhary, N., Salali, G. D., Thompson, J., Mace, R., & Migliano, A. B. (2019). Testing adaptive hypotheses of alloparenting in Agta foragers. *Nature Human Behaviour*, *3*(11). https://doi.org/10.1038/s41562-019-0679-2

Scelza, B. A. (2009). The grandmaternal niche: Critical caretaking among Martu Aborigines. *American Journal of Human Biology*, *21*(4). https://doi.org/10.1002/ajhb.20934
